# Keystone-designed buried de-epithelialized flap: A novel technique for obliterating small to moderately sized dead spaces

**DOI:** 10.1097/MD.0000000000007008

**Published:** 2017-05-26

**Authors:** Hoon Kim, Wan Cheol Ryu, Chi Sun Yoon, Kyu Nam Kim

**Affiliations:** a Department of Plastic and Reconstructive Surgery, Konyang University Hospital, University of Konyang College of Medicine, Myunggok Medical Research Center, Daejeon; b Department of Plastic and Reconstructive Surgery, Ulsan University Hospital, University of Ulsan College of Medicine, Ulsan, Korea.

**Keywords:** reconstructive surgical procedures/methods, surgical dead spaces, surgical flaps

## Abstract

Effective obliteration of dead space after reconstructive surgery facilitates a good cosmetic outcome and prevention of delayed wound healing and recurrent infection.

We evaluated the efficacy of a keystone-designed buried de-epithelialized (KBD) flap for the obliteration of small to moderately sized surgical dead spaces.

We reviewed the medical records of patients who received a KBD flap following removal of a mass or debridement of necrotic tissue from September 2015 to February 2016. The diagnosis, site, dead space dimensions, flap width, drain data, complications, and follow-up duration were recorded.

Twenty-eight KBD flaps were evaluated, including 9 cases of fat necrosis, 7 cases of epidermal cyst, and 12 cases of lipoma. Dead space dimensions ranged from 2 × 1.5 × 1 cm to 10 × 5 × 3 cm, with a mean depth of 2.01 cm. Flap sizes ranged from 2.5 × 1 cm to 11 × 3 cm, with a mean flap width of 2.01 cm. No postoperative complications, such as seroma or hematoma, occurred. The cosmetic results were favorable, and all patients were satisfied with their final outcomes.

The KBD flap is useful for the obliteration of small to moderately sized surgical dead spaces both spatially and physiologically and shows excellent cosmetic outcomes.

## 1 Introduction

The surgical removal of a mass and debridement of necrotic tissue may result in a “dead space” of varying depth and size. Ineffective closure of this cavity can result in a hematoma or seroma, thereby increasing the risk of bacterial infection.^[1,2]^ Thus, obliteration of dead space is crucial to prevent delayed wound healing and wound infection.^[2]^ Effective elimination of dead space also improves the cosmetic outcome by minimizing surgical site contour irregularities. Many reconstructive methods for filling dead space have been used, including various locoregional flap and free flap techniques.^[1]^ Large dead spaces with exposed critical structures must be filled with thick volumetric flaps, such as muscle or fasciocutaneous flaps, which have abundant soft tissue. However, in small or moderately sized dead spaces without exposed structures, volumetric flaps are unnecessary. These cavities may be closed primarily by using undermining skin flaps, but persistent dead space can cause the aforementioned problems. Herein, we present a retrospective review of our experience using a newly described keystone-designed buried de-epithelialized (KBD) flap that we developed for the treatment of small to moderately sized dead spaces after lesion excision or debridement of necrotic tissue. We aimed to determine the efficacy and feasibility of the KBD flap for obliterating these dead spaces.

## 2 Materials and methods

We obtained written informed consent from all patients. The study protocol conformed to the ethical guidelines of the 1975 Declaration of Helsinki as reflected in approval by our Institutional Review Board of Ulsan University Hospital (UUH 2016–02–001).

We retrospectively reviewed the charts of patients who received a KBD flap to obliterate a small to moderately sized dead space following removal of a mass or debridement of necrotic tissue. We defined dead space as a cavity resulting from surgical removal of content that did not contain critical structures (vessels, nerves, or tendons) but would permit body fluid accumulation. We recorded each patient's age and sex, diagnosis, surgical site, surgical procedure, dead space width, length, and depth (cm), flap dimensions, drain data, complications, and follow-up duration in all cases. Three independent plastic surgeons graded the postoperative cosmetic outcome as excellent, good, fair, or poor, according to the Harris 4-stage scale, by comparing preoperative and postoperative clinical photographs. Patient satisfaction was assessed at final follow-up using a scale of 1 to 10, in terms of surgical site contour and scar.

### 2.1 Operative techniques: KBD flap

**Figure 1:**
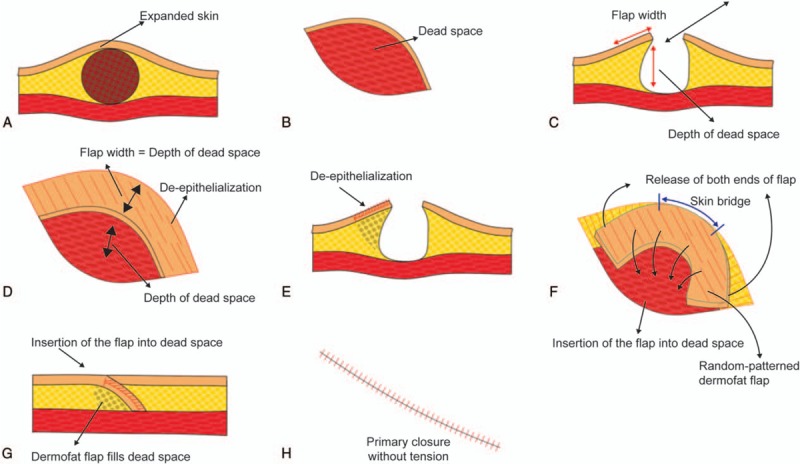
Illustrated summary of the keystone-designed buried de-epithelialized (KBD) flap for obliterating small to moderately sized dead spaces. (A) The lesion usually expands the skin. Following skin incision, excision or debridement is performed. (B, C) The resulting cavity dimensions are measured. (D) The keystone-designed flap is developed at the margin of the ellipse. The flap angle in relation to the margin (0 degree at the tips and 90 degree at the apex) results in a curvilinear flap with a width equal to the dead space depth. (E, F) The flap is de-epithelialized, and both ends are released, forming a random-patterned dermofat flap. (G) The dead space is filled following insertion of the KBD flap. (H) The wound is closed primarily without tension.

Preoperatively, we marked the lesion boundary and the site of the incision based on the relaxed skin tension line (RSTL). An incision was made in the expanded skin overlying the lesion, creating an elliptical surgical field in which excision or debridement was performed (Fig. 1A). After removal of the lesional content, the resulting cavity was measured (Fig. 1B and C). A modified keystone-designed flap was developed along one edge of the ellipse between 0 and 90 degree in relation to its corners, creating a curvilinear flap with a width equal to the depth of the dead space (Fig. 1C and D). The flap was de-epithelialized, and both ends were released forming a random-patterned dermofat flap (Fig. 1E and F). This KBD flap was folded and inserted into the dead space (Fig. 1F and G). The wound was closed primarily with subcutaneous and skin sutures without tension (Fig. 1H). A Silastic drain was inserted, if necessary.

## 3 Results

**Figure 2:**
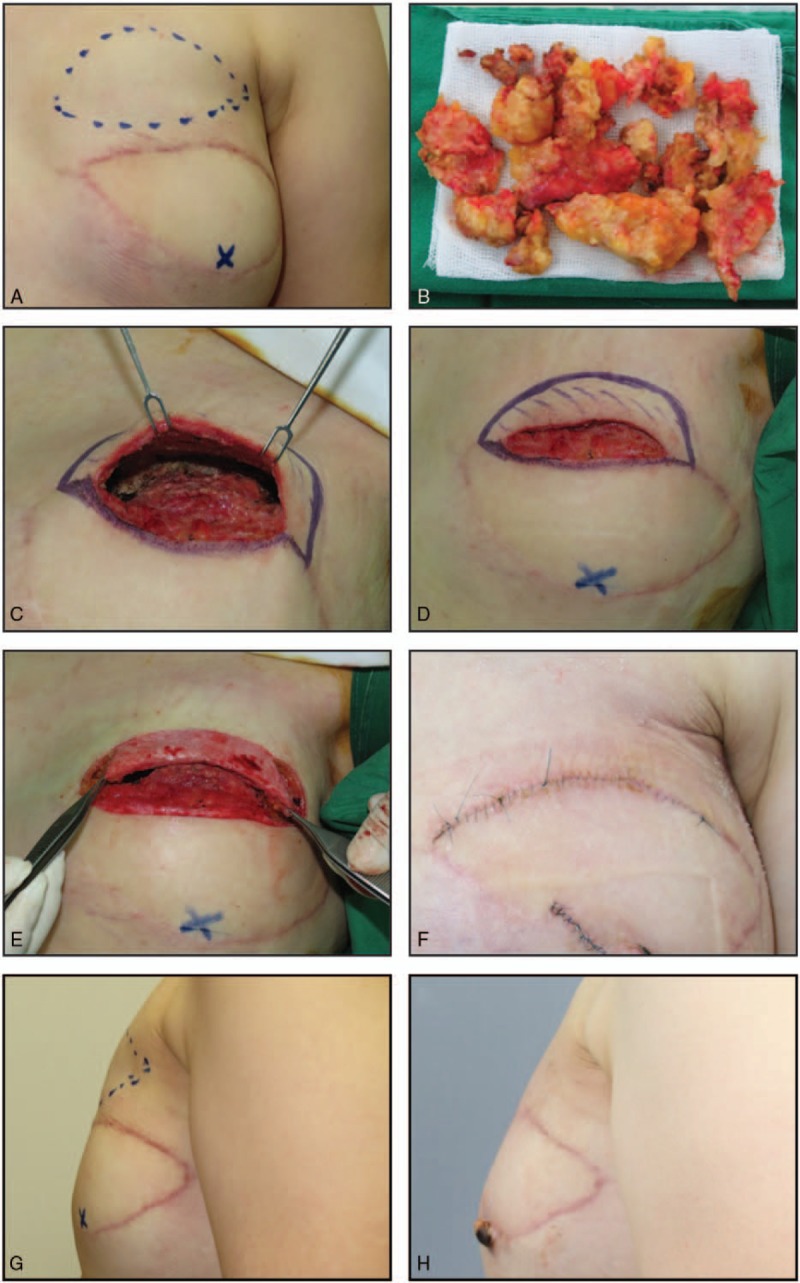
Keystone-designed buried de-epithelialized (KBD) flap after debridement of fat necrosis in a 46-year-old woman following postmastectomy reconstruction with pedicled transverse rectus abdominis myocutaneous flap. (A) The right upper quadrant breast lesion appears as a protrusion measuring approximately 10 × 7 cm. (B) Following incision of the previous scar, necrotic tissue is debrided. (C) The resulting cavity (horizontal length × vertical length × depth, 8 × 5 × 3 cm). (D) The 9 × 3-cm keystone-designed flap site superior to the wound. The width is equal to the dead space depth. (E) The random-patterned dermofat flap following de-epithelialization and release of both flap ends. (F) The wound following primary tension-free closure. (G) Preoperative photograph of the right upper quadrant breast lesion. (H) Postoperative photograph of the breast at 8 months showing a good contour without depression or protrusion.

**Figure 3:**
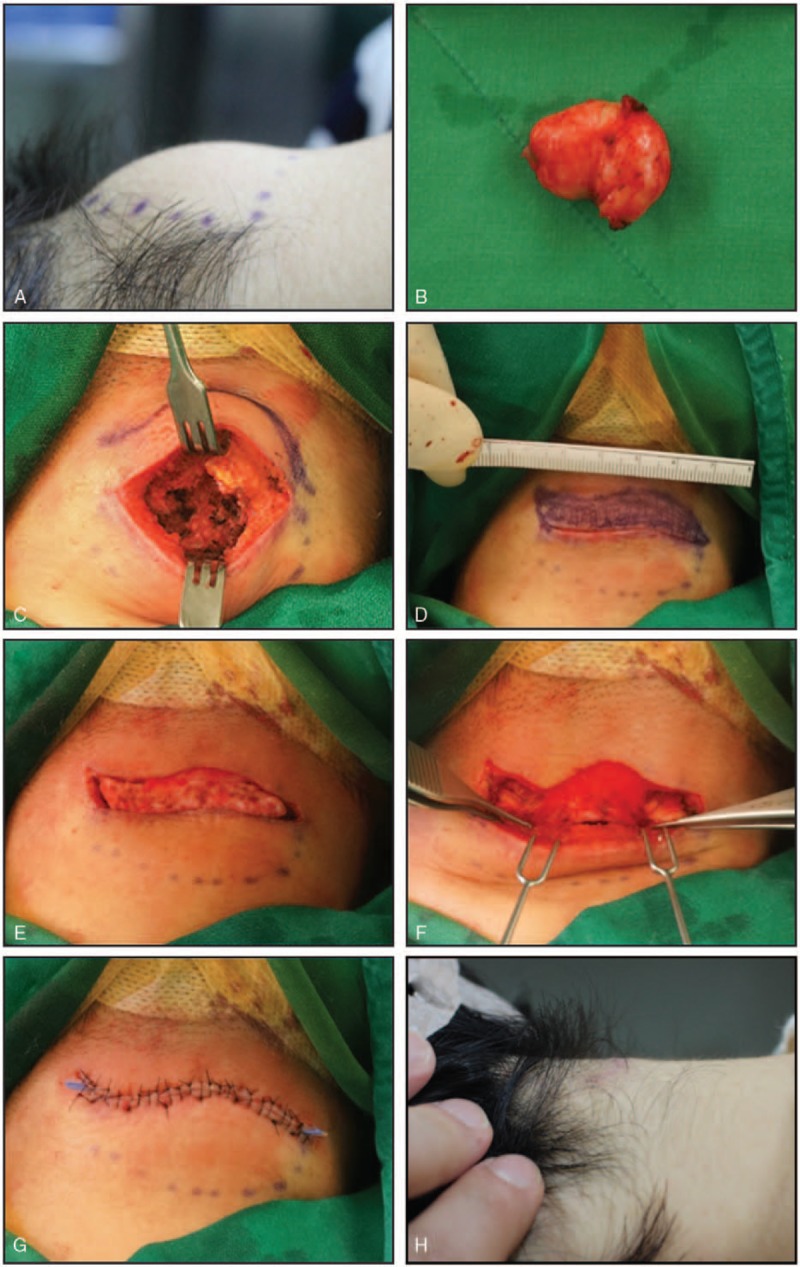
Keystone-designed buried de-epithelialized (KBD) flap after posterior neck lipoma excision in a 46-year-old woman. (A) The posterior neck mass appears as a protrusion measuring approximately 5 × 4 cm. (B) The excised lipoma. (C) The resulting dead space (horizontal length × vertical length × depth, 4 × 3 × 1.5). (D) The 5 × 1.5-cm keystone-designed flap site proximal to the wound. The width is equal to the dead space depth. (E, F) The flap is de-epithelialized, and both ends are released forming a random-patterned dermofat flap. (G) The wound following tension-free closure and Silastic drain placement. (H) Postoperative photograph at 5 months showing good contour with no depression or protrusion.

Patient data are summarized in Table 1. Twenty-eight patients (14 male) aged 28 to 59 years (average, 46.5 years) underwent KBD flap reconstruction during the study period, and each case involved 1 flap. Diagnoses included fat necrosis (9), epidermal cyst (7), and lipoma (12). The dead space volume (horizontal length × vertical length × depth) varied from 2 × 1.5 × 1 cm to 10 × 5 × 3 cm, and the depth varied from 1 to 4 cm (mean depth, 2.01 cm). All cases of fat necrosis occurred as a complication of transverse rectus abdominis myocutaneous flap breast reconstruction. Epidermal cyst sites included the mandible border (1), back (1), cheek (1), and preauricular area (4). Lipoma sites included the lower leg (1), back (6), flank (3), and posterior neck (2). Flap sizes varied from 2.5 × 1 to 11 × 3 cm and widths varied from 1 to 4 cm (mean width, 2.01 cm). Tension-free closure was performed in all cases. A Silastic drain was inserted in 11 cases and removed 1 to 2 days (mean, 1.47 days) postoperatively. No immediate postoperative complications occurred, such as hematoma, seroma, or wound dehiscence, in any patient. During the follow-up (average 6.53 months; range, 5–10 months), no patient experienced a seroma or lesion recurrence. The mean patient satisfaction score was 8.17 ± 0.96 (range, 7–10) (Table 2). Postoperative cosmetic outcomes, evaluated by 3 independent plastic surgeons, were favorable (excellent or good) (Table 2). Photographs illustrating the initial lesion, operative procedures, and postoperative outcomes in 2 cases are shown in Figures 2 and 3.

**Table 1:**
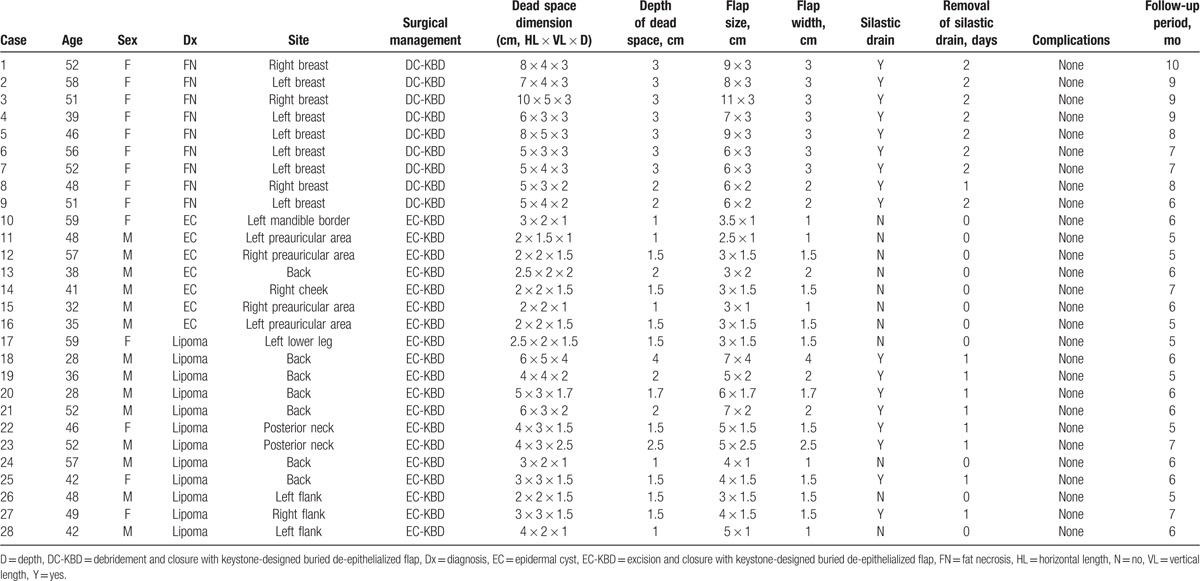
Patient data.

**Table 2:**
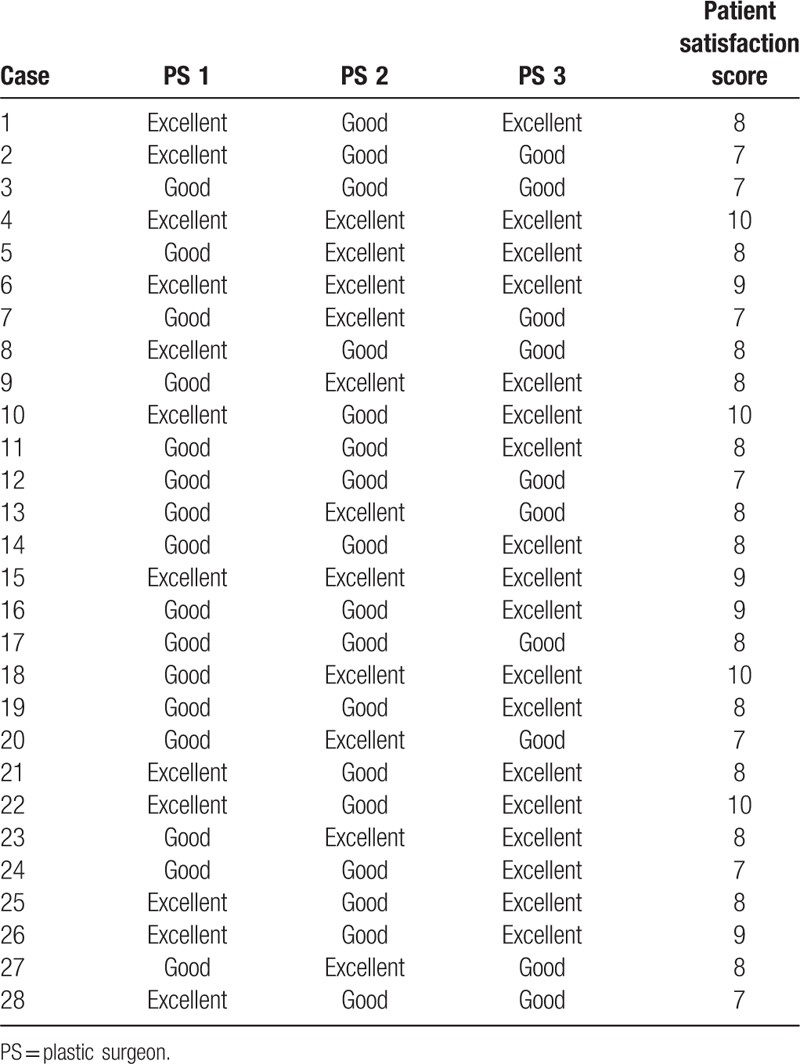
Postoperative cosmetic outcome determined by using Harris 4-stage scale and postoperative satisfaction survey.

## 4 Discussion

**Figure 4:**
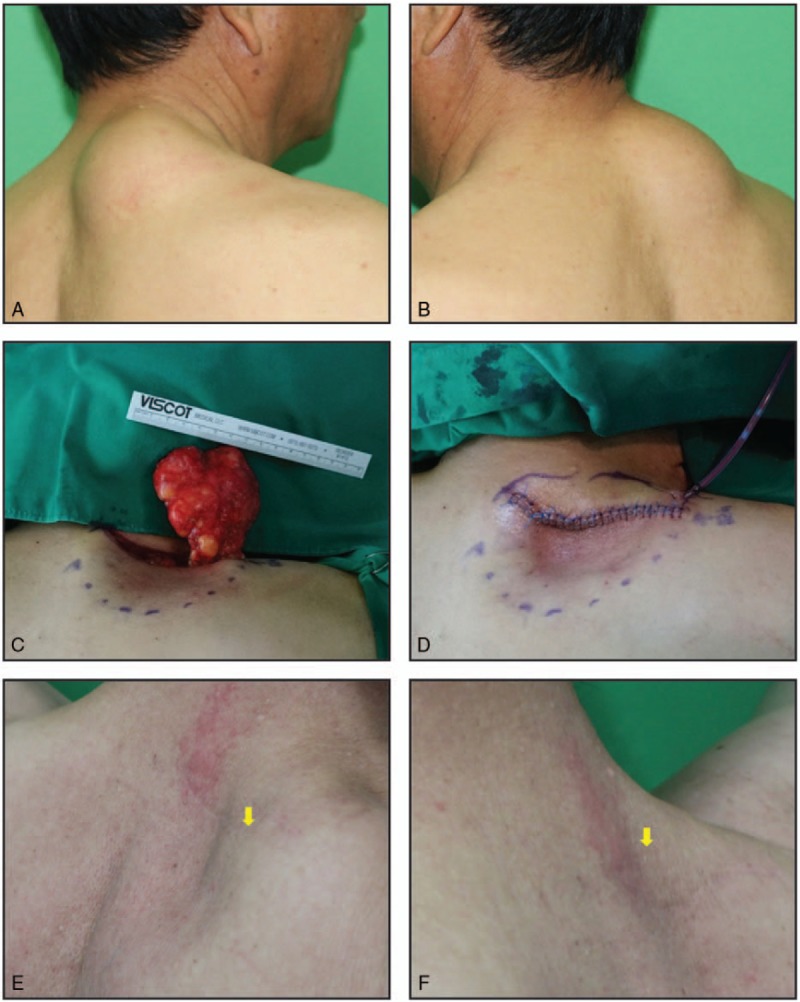
Primary closure after posterior neck lipoma excision. (A, B) A large neck mass presenting in a 53-year-old man. (C) The excised lipoma (approximately 7 × 6 × 3.5 cm). (D) The wound following layer-by-layer closure without filling of the dead space showing a contour depression. (E, F) Postoperative photographs at 8 months showing a persistent skin contour depression (yellow arrow).

Obliteration of dead space is a basic principle of reconstructive surgery. Persistent dead space leads to fluid collection and bacterial growth, resulting in delayed healing and chronic wounds.^[1,2]^ As mentioned earlier, dead spaces may occur if surgical excision sites are not properly filled. Large cavities, such as those occurring after reconstruction of pressure sores or wide excision of malignant skin tumors, require closure using various flap techniques. Muscle,^[3]^ musculocutaneous,^[4,5]^ and fasciocutaneous flaps^[1]^ are the alternatives in such cases. Each has advantages and disadvantages, and the optimal flap should be selected based on the size of the dead space among other factors.^[1]^ Meanwhile, small dead spaces lacking exposure of critical anatomic structures, such as those occurring after the excision of a benign lesion, may be closed using a layer-by-layer closure with skin flap advancement. Although this technique is a simple and efficient means of obliterating dead space, problems may occur. First, inappreciable dead space may remain, especially in large wounds. Second, surface irregularities, such as depressions and dimpling, tend to occur. This may be prevented using layer-by-layer closure with skin flap advancement and eversion of the suture margin in smaller wounds. However, in larger cavities, greater volume deficiency leads to more skin irregularity (Fig. 4A–F). Therefore, volume supplementation is necessary to achieve a good cosmetic outcome. Fat or dermofat grafting^[6]^ is one alternative for filling large spaces. Drawbacks of this method include the need for additional procedures, resorption of the graft, and donor site complications. In this study, we devised the flap and evaluated its suitability as an alternative to these methods.

The keystone design perforator island flap is a multiperforator advancement flap originally described in 2003 by Behan.^[7]^ It has a curvilinear trapezoidal shape reminiscent of the keystone in Roman arches and is constructed over dermatomal segments with a flap width to elliptical defect ratio of 1:1.^[7,8]^ This flap has been described as a combination of 2 opposing V-Y flaps: the initial V-Y advancement at the corners of the keystone flap along the longitudinal axis toward the center and parallel to the defect provides residual laxity within the flap, allowing for translation or advancement of the keystone horizontally into the defect.^[7,9]^ In this study, we developed a keystone flap along the skin margin of the elliptical incision, describing an arc between the corners. The angle of the flap in relation to the skin margin (0 degree at each tip and 90 degree at the apex) resulted in a curvilinear flap with width equal to the dead space depth. We performed de-epithelialization of the flap and released both lateral ends with a full-thickness skin incision leaving a skin bridge along the greater arc of the flap in the manner of the Sydney Melanoma Unit modified keystone flap.^[10]^ The result was a random-patterned dermofat flap consisting of 2 lateral limbs and a greater arc running in a curve parallel to one side of the excision margin. The flap was folded and inserted into the wound beneath the opposite subcutaneous tissue, thus obliterating the dead space. When contrasted with conventional layer-by-layer closure, we think that the KBD flap described here minimizes imperceptible dead space more effectively and may reduce drainage requirements. In our study, 17 of 28 patients required a drain. These drains were removed 1.47 days postoperatively (mean duration) without any complications. We did not use suction drains or pressure garments. We achieved satisfactory results with no complications through the application of only passive drainage (Silastic drains) with the KBD flap.

Seromas, a common postoperative complication, are subcutaneous serous fluid collections arising at previous surgical sites.^[11]^ Although the exact mechanism is incompletely understood, various theories have been proposed to account for their formation, from the disruption of lymphatic and vascular channels to an acute inflammatory process.^[11]^ Chronic and recurrent seromas can impair wound healing, lengthen recovery time, cause patient discomfort, and may require subsequent reoperations.^[11]^ In 1959, Thompson^[12]^ first used the buried dermal flap for the treatment of chronic lymphedema of the lower limb and described that direct anastomoses between the superficial dermal lymphatic plexus in the buried flap and the deep lymph trunks draining the muscular compartment occurred.^[13]^ Later, Nayak and Narayan ^[11]^ demonstrated the usefulness of buried dermal flaps for the treatment of chronic postoperative seromas. Their rationale was that if the seroma recurred, the dermal flap would absorb the seroma fluid via dermal lymphatics.^[11]^ In our study, no immediate postoperative or late seroma occurred in any case. We hypothesize that the KBD flap acts not only by obliterating the dead space physically but also by absorbing the seroma fluid physiologically.

Although the KBD flap described here is a simple and effective technique to obliterate dead space, it does have limitations. First, extension of the scar is inevitable, and the extent is based on the dead space depth. However, the scar occurs on the existing incision line in contrast with other flap and graft techniques. Furthermore, because the incisions were made along the RSTL in all our cases, the scars were also parallel to the RSTL, which might facilitate favorable cosmetic outcomes. Second, this technique may be difficult to apply in areas of excessive skin tension because the flap is parallel to the defect.^[7,10]^ However, the use of an expanded skin flap does not matter in most cases because the underlying lesion causes skin expansion. The major limitation of our study is that it is a nonrandomized, retrospective study without a comparison group; therefore, selection bias and confounding factors are inevitable. A prospective study with a larger sample size and longer follow-up is required to confirm the consistently favorable outcomes we found. We plan to perform a prospective study following the institution of this procedure as a routine elective option in our hospital for the reconstruction of small to moderately sized dead spaces.

In conclusion, the KBD flap is a relatively easy technique (vs. other flap and graft techniques) that, to the best of our knowledge, has not been previously described. We developed this method for the treatment of small to moderately sized dead spaces. The KBD flap successfully obliterates dead space both spatially and physiologically and shows excellent cosmetic results.

## Acknowledgments

The authors have no sources of funding to declare.

## References

[1] Yamauchi T Kiyokawa K Inoue Y V-Y fasciocutaneous flap of the medial thigh including the long saphenous vein for reconstruction of intrapelvic dead space. Scand J Plast Reconstr Surg Hand Surg 2009;43:142–7. 19401944 10.1080/02844310902771657

[2] Oliver RA Lovric V Yu Y Development of a novel model for the assessment of dead-space management in soft tissue. PloS One 2015;10:e0136514. 26305692 10.1371/journal.pone.0136514 PMC4549236

[3] Shibata D Hyland W Busse P Immediate reconstruction of the perineal wound with gracilis muscle flaps following abdominoperineal resection and intraoperative radiation therapy for recurrent carcinoma of the rectum. Ann Surg Oncol 1999;6:33–7. 10030413 10.1007/s10434-999-0033-4

[4] McCraw JB Massey FM Shanklin KD Vaginal reconstruction with gracilis myocutaneous flaps. Plast Reconstr Surg 1976;58:176–83. 781700 10.1097/00006534-197608000-00006

[5] Tobin GR Day TG Vaginal and pelvic reconstruction with distally based rectus abdominis myocutaneous flaps. Plast Reconstr Surg 1988;81:62–73. 2962215 10.1097/00006534-198801000-00012

[6] McNichols CH Hatef DA Cole P Contemporary techniques for the correction of temporal hollowing: augmentation temporoplasty with the classic dermal fat graft. J Craniofac Surg 2012;23:e234–8. 22627443 10.1097/SCS.0b013e31824de5b8

[7] Behan FC The keystone design perforator island flap in reconstructive surgery. ANZ J Surg 2003;73:112–20. 12608972 10.1046/j.1445-2197.2003.02638.x

[8] Behan FC Lo CH Shayan R Perforator territory of the keystone flap–use of the dermatomal roadmap. J Plast Reconstr Aesthet Surg 2009;62:551–3. 10.1016/j.bjps.2008.08.078 19046659

[9] Mohan AT Rammos CK Akhavan AA Evolving concepts of Keystone Perforator Island Flaps (KPIF): principles of perforator anatomy, design modifications, and extended clinical applications. Plast Reconstr Surg 2016;137:1909–20. 26895582 10.1097/PRS.0000000000002228

[10] Moncrieff MD Bowen F Thompson JF Keystone flap reconstruction of primary melanoma excision defects of the leg-the end of the skin graft? Ann Surg Oncol 2008;15:2867–73. 18629589 10.1245/s10434-008-0018-8

[11] Nayak N Narayan D Buried dermal flap for the treatment of chronic postoperative seroma. BMJ Case Rep 2010;2010:pie: bcr0120102668. 10.1136/bcr.01.2010.2668 PMC3029351 22767472

[12] Thompson N Surgical treatment of chronic lymphoedema of the lower limb. With preliminary report of new operation. Br Med J 1962;2:1566–73. 13981190 10.1136/bmj.2.5319.1566 PMC1926791

[13] Thompson N Buried dermal flap operation for chronic lymphedema of the extremities. Ten-year survey of results in 79 cases. Plast Reconstr Surg 1970;45:541–8. 4910556 10.1097/00006534-197006000-00003

